# O059. Predictors of response to botulinum toxin for the treatment of chronic migraine: data from a Headache Center

**DOI:** 10.1186/1129-2377-16-S1-A179

**Published:** 2015-09-28

**Authors:** Raffaele Ornello, Serena Vittoria Lisi, Diana Degan, Cindy Tiseo, Francesca Pistoia, Antonio Carolei, Simona Sacco

**Affiliations:** Department of Neurology, University of L'Aquila, L'Aquila, Italy

## Background

Chronic migraine (CM) is a disabling type of primary headache for which treatment with botulinum toxin type A (BoNT-A) has proved to be effective and safe in reducing headache days and headache-related disability. However, little is known about factors influencing the response to BoNT-A. We aimed to assess which baseline factors predicted a favorable response to BoNT-A in a sample of patients with CM.

## Materials and methods

Consecutive patients admitted to our Regional Headache Referral Center from October 2013 to May 2015, diagnosed with CM and eligible for treatment with BoNT-A, were administered a structured questionnaire investigating baseline characteristics including smoke, alcohol abuse, psychostimulants such as caffeine, arterial hypertension, family history of headaches, headache features (aura, autonomic symptoms, pain intensity assessed through the Visual Analog Scale [VAS], and allodynia), disability (by means of MIDAS and HIT-6 scores), drug abuse, depression (by means of Beck Depression Inventory [BDI]), and anxiety (by means of Generalized Anxiety Disorder [GAD] score). Chi-squared test and Mann-Whitney tests were used for univariate comparisons where appropriate. Logistic regression analysis was used to assess favorable response (≥50% reduction in headache days) to BoNT-A treatment.

## Results

**Figure 1:**
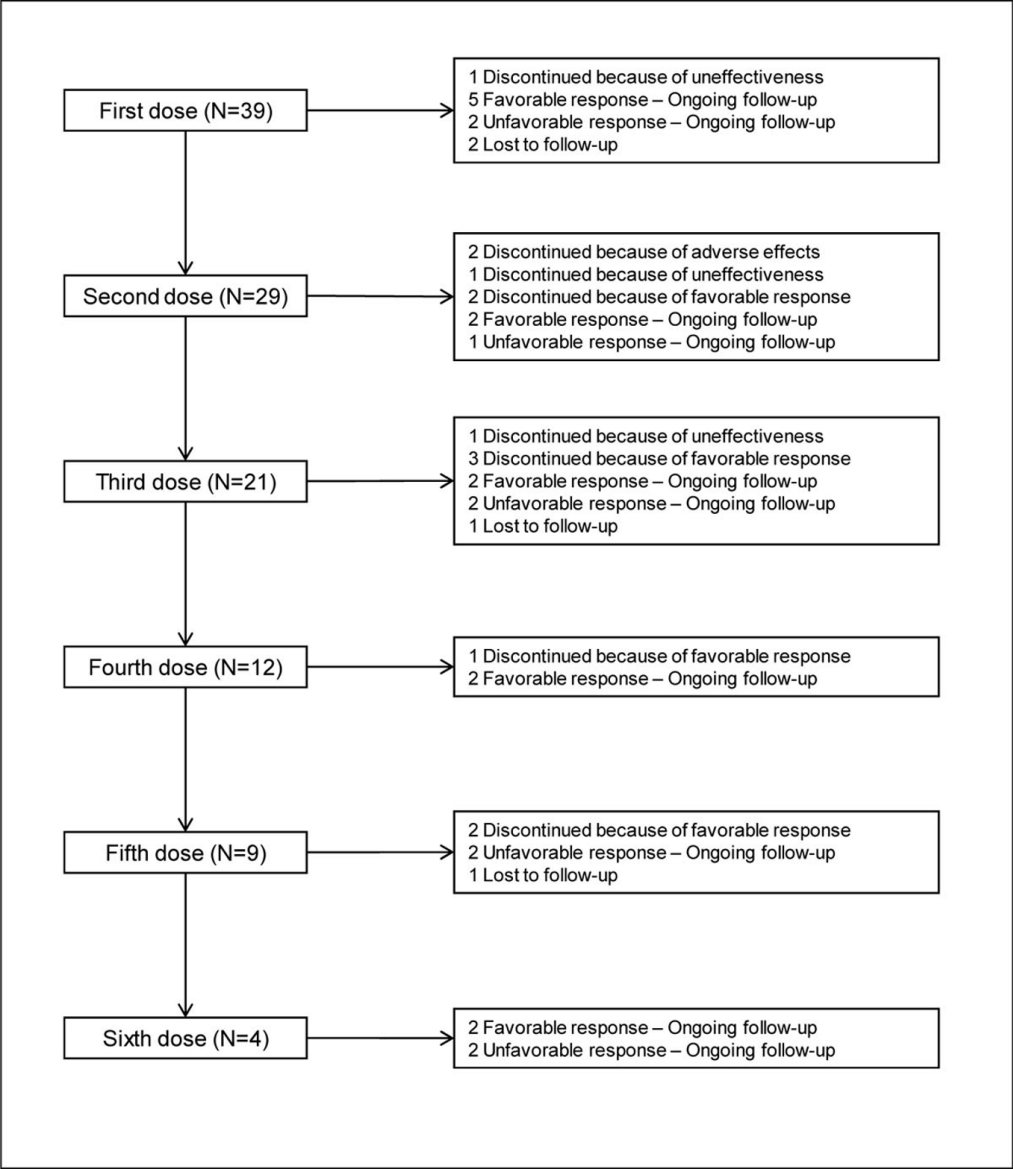

Thirty-nine subjects (32 women and 7 men; mean age ± standard deviation, 48.3±9.9 years) were recruited. Twenty-eight (71.8%) patients were on treatment with triptans, 32 (82.1%) were on preventive treatment, and 22 (56.4%) had drug abuse. One dose of BoNT-A was administered to 10 (25.6%) patients, 2 doses to 8 (20.5%) patients, 3 doses to 9 (23.1%) patients, 4 doses to 3 (7.7%) patients, 5 doses to 5 (12.8%) patients, and 6 doses to 4 (10.3%) patients (Figure 1). A favorable response was reported by 16 (41.0%) patients, while adverse effects were reported by 12 (30.8%) patients, with allergy, muscle tension, numbness, and trapezius atrophy reported by 2 (5.1%) patients each, and pain, orbicularis or frontalis muscle deficit, and headache worsening reported by 1 (2.6%) patient each. Two (5.1%) patients discontinued treatment with BoNT-A because of adverse effects. Distribution of all baseline variables did not differ between subjects with a favorable and a non-favorable response. None of the baseline variables predicted a favorable response to BoNT-A treatment or the development of adverse effects.

## Conclusions

In our case series, baseline variables did not predict a favorable effect or adverse effects from BoNT-A therapy. Our findings suggest that further research is needed to assess predictors of response to that therapy in order to improve management and treatment of CM.

Written informed consent to publish was obtained from the patient(s).

## Conflicts of interest

None.

